# Functions of the Spleen

**Published:** 1884-08-20

**Authors:** 


### FUNCTIONS OF THE SPLEEN.

In the last number of Virchow's Archiv, Professor Alex. Tauber, of Warsaw, gives, as quoted by the Lancet, an excellent review of the theories concerning the functions of the spleen, closing with the results of his own observations.

Thus, in 1833, Tiedemann considered the spleen to be a blood-forming organ, and that after its removal its function was transferred to the thyroid body; an opinion contested by Brucke, who held there was nothing in common between the thyroid and the lymphatic system, to which the spleen belonged. Neumann and Ehrlich doubt that the spleen has any hæmapoietic function. Schiff and Herzen think that the spleen develops a ferment which is necessary to the pancreas in its digestive action, a view disproved by the fact that nutrition is not impaired by removal of the spleen. Schiff goes further by asserting that its removal does not influence, either actually or relatively, the amount of red and white corpuscles, whilst Robin, on the other hand, found in two cases of splenic extirpation in the human subject an increase in the number of leucocytes, and notable variations in the size of the corpuscles. Recently, Winogradaff, of St. Petersburg, found very marked changes in the lymphatic glands, and also in the bone-marrow of animals deprived of the spleen, the former being softer than normal, and containing many free red blood corpuscles. At the German Surgical Congress, held at Dresden in 1882, Dr. Crede showed a man forty-four years of age, from whom he had successfully removed a cystic spleen. Two months after the operation the blood was found to contain a large excess of leucocytes, but three months later their number was nearly normal. Crede had observed in the early weeks after the excision that the thyroid had enlarged in size—an enlargement which persisted to a certain extent. The lymphatic glands did not enlarge. Crede concluded from this and other cases that the splenic function was to convert white into red corpuscles, and that leucocytosis ensues on removal of the organ until the thyroid is enabled to act vicariously.

This idea was further expanded by Zesas, who published the details of experiments on the subject at the end of 1882, from which he concluded that the spleen converted white into red corpuscles, but that other organs also had this power; that increase in the number of white corpuscles and diminution in the red occur upon extirpation of the spleen until the above function is taken on by another organ, and that this property of acting vicariously to the spleen resides in the thyroid as well as in the bronchial and mesenteric glands. The lymphatic glands alone are incapable of supplying the needs of the organism in this respect, so that simultaneous removal of the spleen and thyroid is incompatible with life, this last being based on the issue of a single experiment.

Tauber remarks that practical surgery now affords more means of arriving at a conclusion, since more than fifty cases of extirpation of the spleen are on record. In 1882, previous to performing this operation for cystic degeneration of the organ, he made many experiments on the lower animals, and was thereby convinced that excision of the healthy spleen could be performed without injury to the animal's health. He practiced excisions of the spleen and of the thyroid in fifteen animals, in some removing one of these organs, in others both, and examined the blood in each case. Six of these animals died, one from the operation itself, another from acute peritonitis, the others from weakness within three weeks of the operation, after the wounds had cicatrized; post-mortem examination showing marked congestion of the liver, lungs and lymphatic glands, and intestinal hæmorrhages. He points out that the thyroid is very often entirely wanting in the domestic animals, and yet in such animals splenectomy is as well borne as in those having a well-developed thyroid. These experiments, then, do not support the doctrines advanced by Tiedemann, Crede and Zesas as to an interchange of function between these organs. Nor did Rœher, in his numerous cases of thyroid excision in the human subject, meet with one case in which the spleen became subsequently enlarged.

The deductions Tauber draws from these experiments are:

1. That the spleen must be regarded as one of the main reservoirs of the blood. Its removal, therefore, exerts a great influence on the circulation, as seen in the occurrence, shortly after the excision, of congestion of the liver, kidneys, and especially of the lymphatic glands.

2. No physiological affinity exists between the thyroid and the spleen.

3. An animal of middle age bears splenotomy much better than an old animal, hæmorrhages being liable to occur in the latter.

4. Animals deprived of the spleen can bear living young in whom the spleen is present.

5. The removal of the spleen does not impair the digestive function or nutrition.

6. After removal of the spleen the animal becomes anæmic. The relative and positive number of white corpuscles notably increases, whilst the size and number of the red corpuscles diminish.—*Journal Med. Progress.*

